# Crystal structure of 15-(naphthalen-1-yl)-7,7a,8,9,10,11-hexa­hydro-6a,12a-(methano­epoxy­methano)­indolizino[2,3-*c*]quinoline-6,13(5*H*)-dione

**DOI:** 10.1107/S2056989015002017

**Published:** 2015-02-07

**Authors:** M. P. Savithri, M. Suresh, R. Raghunathan, R. Raja, A. SubbiahPandi

**Affiliations:** a Department of Physics, Queen Mary’s College (Autonomous), Chennai 600 004, India; b Department of Organic Chemistry, University of Madras, Guindy Campus, Chennai 600 025, India; c Department of Physics, Presidency College (Autonomous), Chennai 600 005, India

**Keywords:** crystal structure, quinoline, pyrrolidine, hydrogen bonds

## Abstract

In the title compound, C_27_H_24_N_2_O_3_, the dihedral angle between the mean planes of the di­hydro­furan and 3,4-di­hydro­quinoline ring systems is 70.65 (9)°. The di­hydro­furan ring adopts an envelope conformation with the C atom adjacent to the methyl­ene C atom of the pyrrolidine ring as the flap. The five-membered pyrrolidine ring adopts a twist conformation on the N—C(tetra­substituted) bond. In the crystal, mol­ecules are linked *via* pairs of N—H⋯O hydrogen bonds, forming inversion dimers with an *R*_2_^2^(8) ring motif. The dimers are linked *via* pairs of C—H⋯O hydrogen bonds, forming ribbons enclosing *R*_2_^2^(12) ring motifs lying in a plane parallel to (01-1).

## Related literature   

For general background to quinoline and pyrrolidine derivatives, see: Padwa *et al.* (1999 ▸). For a related structure, see: Govindan *et al.* (2014 ▸).
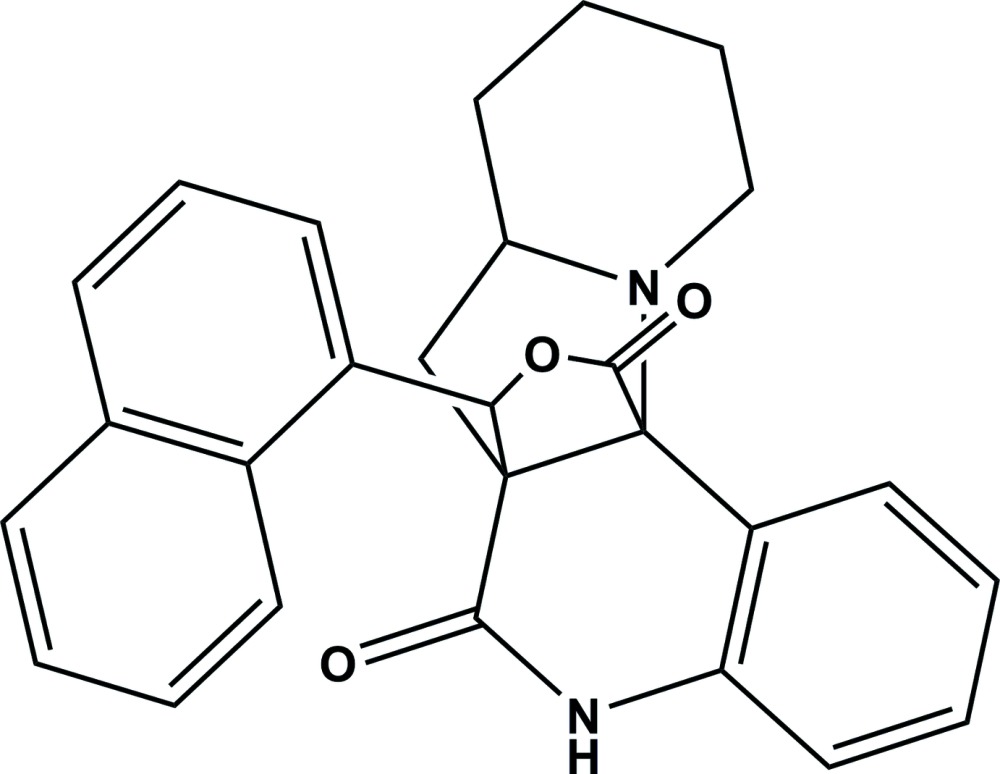


## Experimental   

### Crystal data  

C_27_H_24_N_2_O_3_
*M* *_r_* = 424.49
Triclinic, 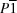

*a* = 9.4184 (3) Å
*b* = 9.8804 (4) Å
*c* = 12.5401 (5) Å
α = 95.341 (2)°
β = 107.535 (2)°
γ = 99.940 (2)°
*V* = 1082.87 (7) Å^3^
*Z* = 2
Mo *K*α radiation
μ = 0.09 mm^−1^
*T* = 293 K
0.35 × 0.30 × 0.30 mm

### Data collection  

Bruker Kappa APEXII CCD diffractometer
Absorption correction: multi-scan (*SADABS*; Bruker, 2004 ▸) *T*_min_ = 0.971, *T*_max_ = 0.975
28346 measured reflections
3817 independent reflections
3007 reflections with *I* > 2σ(*I*)
*R*_int_ = 0.023

### Refinement  

*R*[*F* ^2^ > 2σ(*F* ^2^)] = 0.040
*wR*(*F* ^2^) = 0.157
*S* = 0.87
3817 reflections
293 parameters
H atoms treated by a mixture of independent and constrained refinement
Δρ_max_ = 0.17 e Å^−3^
Δρ_min_ = −0.21 e Å^−3^

### 

Data collection: *APEX2* (Bruker, 2004 ▸); cell refinement: *APEX2* and *SAINT* (Bruker, 2004 ▸); data reduction: *SAINT* and *XPREP* (Bruker, 2004 ▸); program(s) used to solve structure: *SHELXS97* (Sheldrick, 2008 ▸); program(s) used to refine structure: *SHELXL97* (Sheldrick, 2008 ▸); molecular graphics: *ORTEP-3 for Windows* (Farrugia, 2012 ▸); software used to prepare material for publication: *SHELXL97* and *PLATON* (Spek, 2009 ▸).

## Supplementary Material

Crystal structure: contains datablock(s) global, I. DOI: 10.1107/S2056989015002017/su5066sup1.cif

Structure factors: contains datablock(s) I. DOI: 10.1107/S2056989015002017/su5066Isup2.hkl

Supporting information file. DOI: 10.1107/S2056989015002017/su5066Isup3.cml

**Fig. 1.**
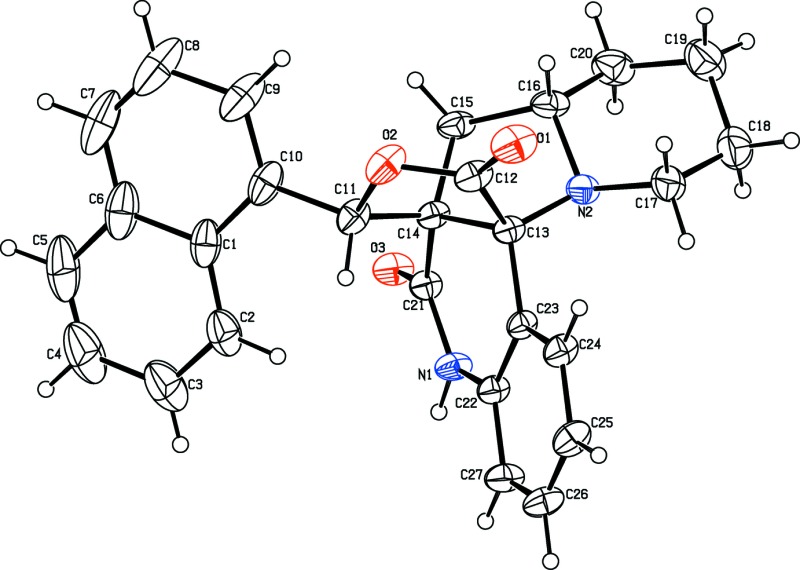
The molecular structure of the title compound, showing the atom labelling. Displacement ellipsoids are drawn at the 30% probability level.

**Fig. 2.**
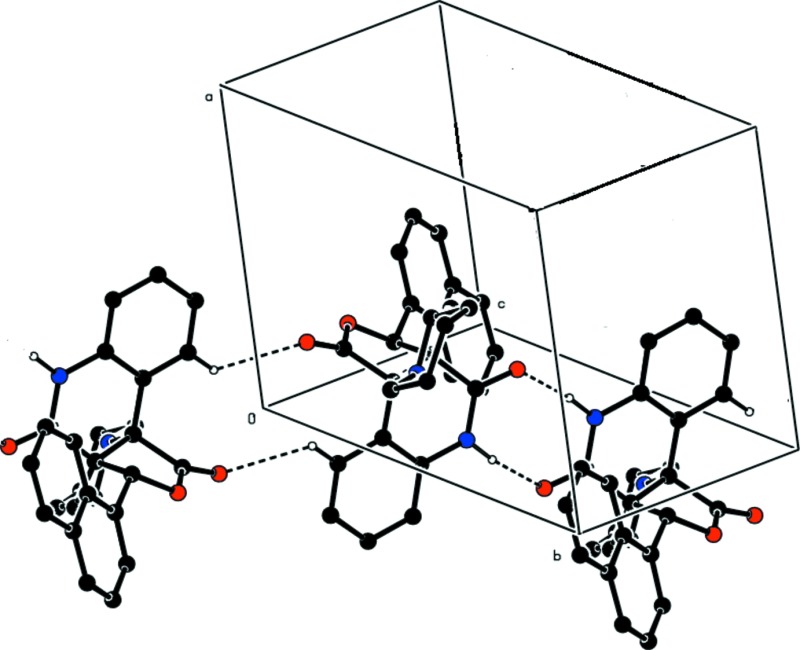
A partial view along the b axis of the crystal packing of the title compound. The hydrogen bonds are shown as dashed lines (see Table 1 for details).

**Fig. 3.**
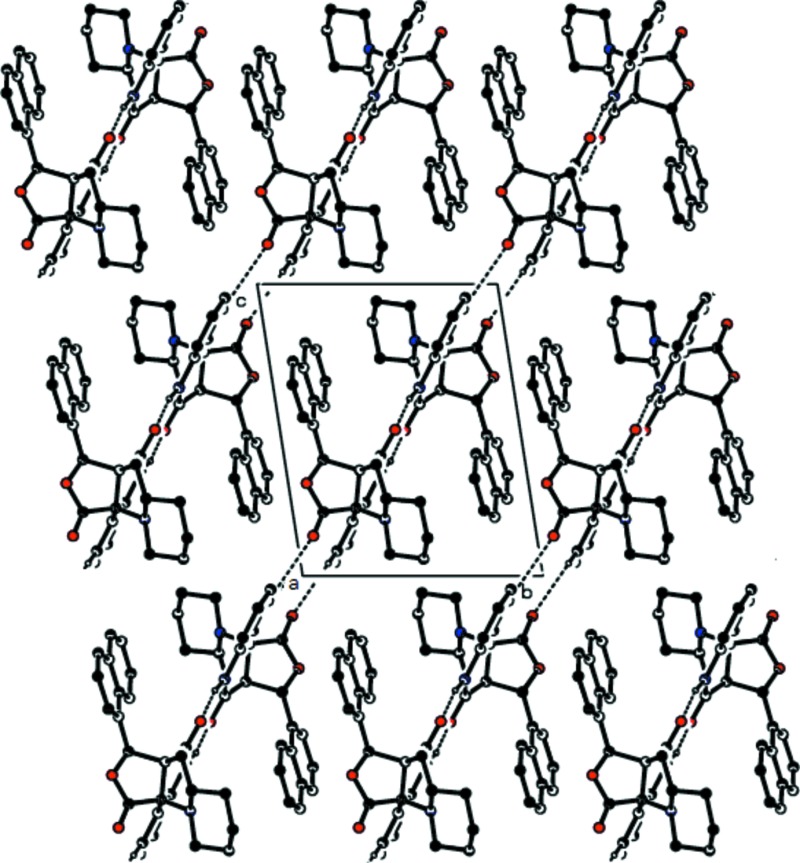
The molecular packing viewed along the a axis. Dashed lines shows the intermolecular C—H···O and N—H···O hydrogen bonds (see Table 1 for details).

. DOI: 10.1107/S2056989015002017/su5066fig1.tif
The mol­ecular structure of the title compound, showing the atom labelling. Displacement ellipsoids are drawn at the 30% probability level.

b . DOI: 10.1107/S2056989015002017/su5066fig2.tif
A partial view along the *b* axis of the crystal packing of the title compound. The hydrogen bonds are shown as dashed lines (see Table 1 for details).

a . DOI: 10.1107/S2056989015002017/su5066fig3.tif
The mol­ecular packing viewed along the *a* axis. Dashed lines shows the inter­molecular C—H⋯O and N—H⋯O hydrogen bonds (see Table 1 for details).

CCDC reference: 1046441

Additional supporting information:  crystallographic information; 3D view; checkCIF report

## Figures and Tables

**Table 1:** Hydrogen-bond geometry (, )

*D*H*A*	*D*H	H*A*	*D* *A*	*D*H*A*
N1H1*A*O3^i^	0.94(3)	1.92(3)	2.8413(19)	167(2)
C24H24O1^ii^	0.93	2.59	3.268(2)	131

Symmetry codes: (i) 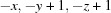; (ii) 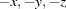.

## supplementary crystallographic information

### S1. Structural commentary

A large number of natural products contain the quinoline and indole heterocycles, and are found in numerous commercial products, including pharmaceuticals, fragrances and dyes (Padwa *et al.*, 1999). In view of the above importance we have synthesized the title compound and report herein on its crystal structure.

The molecular structure of the title molecule is shown in Fig. 1. The furan ring system has an envelope conformation with atom C14 as the flap. The quinoline ring adopts a planar conformation with a maximum deviation of 0.326 (2) Å for the spiro C atom, C14. The five-membered pyrrolidine ring (N2/C13–C16) is twisted on N2—C13. The sum of the bond angles around atom N2 of the o­cta­hydro­indolizine ring is 338.61° and for N1 of the quinoline ring it is 359.71°, confirming the *sp*^3^ and *sp*^2^ hybridization, respectively.

In the crystal, molecules are linked by two pairs of N1—H1A···O3, C24—H24···O1 hydrogen bonds (Table 1), forming two inversion dimers and containing two R^2^_2_(8), R^2^_2_(12) ring motifs, respectively; see Fig. 2. In the crystal structure, inter­molecular C24—H24···O1, N1—H1A···O3 hydrogen bonds link the molecules into ribbons lying parallel to the (011; Fig. 3 and Table 1.

### S2. Synthesis and crystallization

A mixture of methyl 2-((hydroxyl(naphthalene-2-yl) methyl) acrylate (1 mmol), isatin (1.1 mmol) and pipecolic acid (1.1 mmol) was placed in a round bottom flask and melted at 180°C until completion of the reaction was evidenced by TLC analysis. After completion of the reaction, the crude product was washed with 5 ml of ethyl­acetate and hexane mixture (1:4 ratio) which successfully provided the pure product as colorless solid. The product was dissolved in ethyl acetate and heated for two minutes. The resulting solution was subjected to crystallization by slow evaporation of the solvent for 48 hours resulting in the formation of single crystals.

### S3. Refinement

Crystal data, data collection and structure refinement details are summarized in Table 1. All H atoms were fixed geometrically and allowed to ride on their parent C atoms, with C—H distances fixed in the range 0.93-0.98 Å with *U*_iso_ (H) = 1.5*U*_eq_(C) for methyl H 1.2*U*_eq_(C) for other H atoms.

### Crystal data

**Table:**

C_27_H_24_N_2_O_3_	*Z* = 2
*M**_r_* = 424.49	*F*(000) = 448
Triclinic, *P*1	*D*_x_ = 1.302 Mg m^−^^3^
Hall symbol: -P 1	Mo *K*α radiation, λ = 0.71073 Å
*a* = 9.4184 (3) Å	Cell parameters from 3817 reflections
*b* = 9.8804 (4) Å	θ = 1.7–25.0°
*c* = 12.5401 (5) Å	µ = 0.09 mm^−^^1^
α = 95.341 (2)°	*T* = 293 K
β = 107.535 (2)°	Block, colourless
γ = 99.940 (2)°	0.35 × 0.30 × 0.30 mm
*V* = 1082.87 (7) Å^3^	

### Data collection

**Table:**

Bruker Kappa APEXII CCD diffractometer	3817 independent reflections
Radiation source: fine-focus sealed tube	3007 reflections with *I* > 2σ(*I*)
Graphite monochromator	*R*_int_ = 0.023
ω and φ scans	θ_max_ = 25.0°, θ_min_ = 1.7°
Absorption correction: multi-scan (*SADABS*; Bruker, 2004)	*h* = −11→11
*T*_min_ = 0.971, *T*_max_ = 0.975	*k* = −11→11
28346 measured reflections	*l* = −14→14

### Refinement

**Table:**

Refinement on *F*^2^	Primary atom site location: structure-invariant direct methods
Least-squares matrix: full	Secondary atom site location: difference Fourier map
*R*[*F*^2^ > 2σ(*F*^2^)] = 0.040	Hydrogen site location: inferred from neighbouring sites
*wR*(*F*^2^) = 0.157	H atoms treated by a mixture of independent and constrained refinement
*S* = 0.87	*w* = 1/[σ^2^(*F*_o_^2^) + (0.1054*P*)^2^ + 0.6313*P*] where *P* = (*F*_o_^2^ + 2*F*_c_^2^)/3
3817 reflections	(Δ/σ)_max_ < 0.001
293 parameters	Δρ_max_ = 0.17 e Å^−^^3^
0 restraints	Δρ_min_ = −0.21 e Å^−^^3^

### Special details

**Table:**

Geometry. All esds (except the esd in the dihedral angle between two l.s. planes) are estimated using the full covariance matrix. The cell esds are taken into account individually in the estimation of esds in distances, angles and torsion angles; correlations between esds in cell parameters are only used when they are defined by crystal symmetry. An approximate (isotropic) treatment of cell esds is used for estimating esds involving l.s. planes.
Refinement. Refinement of F^2^ against ALL reflections. The weighted R-factor wR and goodness of fit S are based on F^2^, conventional R-factors R are based on F, with F set to zero for negative F^2^. The threshold expression of F^2^ > 2sigma(F^2^) is used only for calculating R-factors(gt) etc. and is not relevant to the choice of reflections for refinement. R-factors based on F^2^ are statistically about twice as large as those based on F, and R- factors based on ALL data will be even larger.

### Fractional atomic coordinates and isotropic or equivalent isotropic displacement parameters (Å^2^)

**Table:**

	*x*	*y*	*z*	*U*_iso_*/*U*_eq_	
N1	−0.03845 (17)	0.39247 (17)	0.35495 (13)	0.0396 (4)	
O3	0.17604 (14)	0.48033 (14)	0.50098 (11)	0.0464 (4)	
N2	0.20514 (17)	0.37970 (17)	0.19482 (12)	0.0406 (4)	
O2	0.20942 (17)	0.07713 (15)	0.31755 (11)	0.0536 (4)	
C13	0.12431 (19)	0.26604 (18)	0.23296 (13)	0.0340 (4)	
O1	0.19536 (18)	0.07035 (17)	0.13733 (12)	0.0619 (5)	
C22	−0.12077 (19)	0.30793 (18)	0.25108 (14)	0.0357 (4)	
C21	0.10973 (19)	0.40178 (18)	0.41158 (14)	0.0351 (4)	
C24	−0.1323 (2)	0.1598 (2)	0.08548 (15)	0.0422 (5)	
H24	−0.0838	0.1149	0.0427	0.051*	
C11	0.1637 (2)	0.16082 (19)	0.39818 (15)	0.0427 (5)	
H11	0.0541	0.1284	0.3826	0.051*	
C14	0.18956 (19)	0.30545 (18)	0.36204 (13)	0.0346 (4)	
C23	−0.04632 (19)	0.24314 (18)	0.18736 (14)	0.0338 (4)	
C16	0.3652 (2)	0.3986 (2)	0.26462 (16)	0.0446 (5)	
H16	0.4082	0.3245	0.2370	0.054*	
C25	−0.2888 (2)	0.1423 (2)	0.04654 (17)	0.0494 (5)	
H25	−0.3453	0.0870	−0.0225	0.059*	
C26	−0.3607 (2)	0.2069 (2)	0.11008 (18)	0.0528 (5)	
H26	−0.4662	0.1948	0.0841	0.063*	
C15	0.3561 (2)	0.3777 (2)	0.38191 (15)	0.0445 (5)	
H15A	0.3823	0.4663	0.4310	0.053*	
H15B	0.4252	0.3199	0.4164	0.053*	
C27	−0.2778 (2)	0.2894 (2)	0.21180 (18)	0.0482 (5)	
H27	−0.3274	0.3329	0.2544	0.058*	
C17	0.1838 (2)	0.3772 (3)	0.07473 (16)	0.0536 (5)	
H17A	0.0762	0.3627	0.0325	0.064*	
H17B	0.2234	0.3016	0.0472	0.064*	
C12	0.1767 (2)	0.1274 (2)	0.21866 (15)	0.0434 (5)	
C1	0.1724 (3)	0.1607 (2)	0.60132 (17)	0.0569 (6)	
C19	0.4345 (3)	0.5443 (3)	0.1301 (2)	0.0723 (7)	
H19A	0.4838	0.6361	0.1231	0.087*	
H19B	0.4842	0.4771	0.1027	0.087*	
C18	0.2675 (3)	0.5145 (3)	0.0586 (2)	0.0695 (7)	
H18A	0.2209	0.5885	0.0796	0.083*	
H18B	0.2592	0.5123	−0.0206	0.083*	
C2	0.0310 (3)	0.1993 (3)	0.5812 (2)	0.0656 (7)	
H2	−0.0232	0.2101	0.5082	0.079*	
C20	0.4522 (3)	0.5368 (3)	0.2539 (2)	0.0614 (6)	
H20A	0.5591	0.5482	0.2970	0.074*	
H20B	0.4139	0.6114	0.2843	0.074*	
C10	0.2439 (3)	0.1421 (2)	0.51715 (17)	0.0552 (6)	
C6	0.2489 (4)	0.1394 (3)	0.71308 (19)	0.0754 (8)	
C5	0.1817 (5)	0.1622 (3)	0.7981 (2)	0.0969 (12)	
H5	0.2310	0.1486	0.8714	0.116*	
C3	−0.0285 (4)	0.2215 (3)	0.6662 (2)	0.0888 (9)	
H3	−0.1213	0.2491	0.6510	0.107*	
C9	0.3821 (3)	0.1050 (3)	0.5444 (2)	0.0792 (9)	
H9	0.4289	0.0949	0.4895	0.095*	
C7	0.3883 (5)	0.0989 (3)	0.7356 (2)	0.0964 (12)	
H7	0.4370	0.0830	0.8083	0.116*	
C8	0.4544 (4)	0.0820 (3)	0.6556 (3)	0.1010 (12)	
H8	0.5477	0.0552	0.6733	0.121*	
C4	0.0487 (5)	0.2030 (4)	0.7754 (3)	0.1065 (13)	
H4	0.0078	0.2191	0.8331	0.128*	
H1A	−0.091 (3)	0.440 (2)	0.393 (2)	0.059 (6)*	

### Atomic displacement parameters (Å^2^)

**Table:**

	*U*^11^	*U*^22^	*U*^33^	*U*^12^	*U*^13^	*U*^23^
N1	0.0337 (8)	0.0502 (9)	0.0332 (8)	0.0164 (7)	0.0089 (6)	−0.0094 (7)
O3	0.0422 (7)	0.0548 (8)	0.0349 (7)	0.0115 (6)	0.0082 (6)	−0.0179 (6)
N2	0.0341 (8)	0.0564 (10)	0.0288 (8)	0.0092 (7)	0.0095 (6)	−0.0019 (7)
O2	0.0661 (9)	0.0510 (8)	0.0385 (8)	0.0316 (7)	0.0030 (7)	−0.0080 (6)
C13	0.0320 (9)	0.0450 (10)	0.0234 (8)	0.0132 (7)	0.0067 (7)	−0.0056 (7)
O1	0.0664 (10)	0.0727 (10)	0.0443 (8)	0.0301 (8)	0.0147 (7)	−0.0206 (7)
C22	0.0324 (9)	0.0414 (9)	0.0305 (9)	0.0107 (7)	0.0068 (7)	−0.0019 (7)
C21	0.0357 (9)	0.0398 (9)	0.0282 (9)	0.0101 (7)	0.0097 (7)	−0.0048 (7)
C24	0.0429 (10)	0.0480 (11)	0.0297 (9)	0.0134 (8)	0.0042 (8)	−0.0048 (8)
C11	0.0524 (11)	0.0426 (10)	0.0297 (9)	0.0186 (8)	0.0064 (8)	−0.0029 (7)
C14	0.0318 (9)	0.0452 (10)	0.0233 (8)	0.0139 (7)	0.0039 (7)	−0.0064 (7)
C23	0.0330 (9)	0.0387 (9)	0.0265 (8)	0.0104 (7)	0.0052 (7)	−0.0006 (7)
C16	0.0314 (9)	0.0609 (12)	0.0380 (10)	0.0119 (8)	0.0093 (8)	−0.0076 (9)
C25	0.0440 (11)	0.0504 (11)	0.0382 (10)	0.0071 (9)	−0.0044 (8)	−0.0053 (9)
C26	0.0317 (10)	0.0616 (13)	0.0539 (12)	0.0101 (9)	−0.0007 (9)	0.0017 (10)
C15	0.0316 (9)	0.0626 (12)	0.0345 (10)	0.0145 (8)	0.0052 (8)	−0.0068 (8)
C27	0.0340 (10)	0.0602 (12)	0.0491 (11)	0.0155 (9)	0.0113 (8)	−0.0013 (9)
C17	0.0490 (11)	0.0771 (15)	0.0322 (10)	0.0095 (10)	0.0128 (9)	0.0046 (10)
C12	0.0402 (10)	0.0516 (11)	0.0327 (10)	0.0176 (8)	0.0040 (8)	−0.0111 (8)
C1	0.0829 (16)	0.0431 (11)	0.0329 (11)	0.0028 (11)	0.0078 (10)	0.0049 (8)
C19	0.0646 (15)	0.0815 (17)	0.0676 (16)	−0.0036 (13)	0.0276 (13)	0.0107 (13)
C18	0.0693 (15)	0.0873 (18)	0.0520 (14)	0.0087 (13)	0.0214 (12)	0.0196 (13)
C2	0.0790 (17)	0.0698 (15)	0.0427 (12)	−0.0030 (13)	0.0229 (11)	0.0061 (11)
C20	0.0452 (12)	0.0704 (15)	0.0592 (14)	−0.0003 (10)	0.0137 (10)	−0.0018 (11)
C10	0.0737 (15)	0.0474 (11)	0.0347 (11)	0.0220 (10)	−0.0004 (10)	0.0013 (9)
C6	0.116 (2)	0.0507 (13)	0.0370 (12)	−0.0002 (14)	0.0025 (13)	0.0049 (10)
C5	0.149 (3)	0.082 (2)	0.0337 (14)	−0.018 (2)	0.0167 (17)	0.0072 (12)
C3	0.095 (2)	0.105 (2)	0.0605 (16)	−0.0118 (17)	0.0393 (16)	0.0013 (15)
C9	0.096 (2)	0.0807 (18)	0.0517 (14)	0.0534 (16)	−0.0060 (13)	0.0000 (12)
C7	0.147 (3)	0.0656 (17)	0.0456 (15)	0.0313 (19)	−0.0193 (18)	0.0083 (13)
C8	0.131 (3)	0.090 (2)	0.0613 (18)	0.066 (2)	−0.0203 (18)	−0.0001 (15)
C4	0.132 (3)	0.123 (3)	0.0484 (17)	−0.026 (3)	0.0393 (19)	−0.0020 (17)

### Geometric parameters (Å, º)

**Table:**

N1—C21	1.344 (2)	C15—H15B	0.9700
N1—C22	1.400 (2)	C27—H27	0.9300
N1—H1A	0.94 (3)	C17—C18	1.510 (3)
O3—C21	1.224 (2)	C17—H17A	0.9700
N2—C13	1.447 (2)	C17—H17B	0.9700
N2—C17	1.455 (2)	C1—C2	1.405 (4)
N2—C16	1.466 (2)	C1—C6	1.423 (3)
O2—C12	1.348 (2)	C1—C10	1.427 (3)
O2—C11	1.456 (2)	C19—C18	1.519 (4)
C13—C23	1.503 (2)	C19—C20	1.521 (3)
C13—C14	1.533 (2)	C19—H19A	0.9700
C13—C12	1.547 (2)	C19—H19B	0.9700
O1—C12	1.193 (2)	C18—H18A	0.9700
C22—C27	1.384 (3)	C18—H18B	0.9700
C22—C23	1.392 (2)	C2—C3	1.363 (4)
C21—C14	1.508 (2)	C2—H2	0.9300
C24—C25	1.380 (3)	C20—H20A	0.9700
C24—C23	1.384 (2)	C20—H20B	0.9700
C24—H24	0.9300	C10—C9	1.365 (4)
C11—C10	1.500 (3)	C6—C7	1.394 (5)
C11—C14	1.541 (3)	C6—C5	1.414 (5)
C11—H11	0.9800	C5—C4	1.340 (5)
C14—C15	1.541 (3)	C5—H5	0.9300
C16—C20	1.506 (3)	C3—C4	1.390 (5)
C16—C15	1.530 (3)	C3—H3	0.9300
C16—H16	0.9800	C9—C8	1.414 (4)
C25—C26	1.371 (3)	C9—H9	0.9300
C25—H25	0.9300	C7—C8	1.343 (5)
C26—C27	1.374 (3)	C7—H7	0.9300
C26—H26	0.9300	C8—H8	0.9300
C15—H15A	0.9700	C4—H4	0.9300
			
C21—N1—C22	125.41 (15)	N2—C17—C18	108.53 (18)
C21—N1—H1A	115.6 (14)	N2—C17—H17A	110.0
C22—N1—H1A	118.7 (15)	C18—C17—H17A	110.0
C13—N2—C17	120.05 (15)	N2—C17—H17B	110.0
C13—N2—C16	105.17 (14)	C18—C17—H17B	110.0
C17—N2—C16	113.39 (15)	H17A—C17—H17B	108.4
C12—O2—C11	109.27 (14)	O1—C12—O2	121.41 (18)
N2—C13—C23	114.84 (14)	O1—C12—C13	128.67 (19)
N2—C13—C14	102.34 (14)	O2—C12—C13	109.83 (14)
C23—C13—C14	113.67 (14)	C2—C1—C6	117.6 (2)
N2—C13—C12	114.32 (14)	C2—C1—C10	124.17 (19)
C23—C13—C12	110.67 (14)	C6—C1—C10	118.2 (3)
C14—C13—C12	99.78 (13)	C18—C19—C20	111.0 (2)
C27—C22—C23	119.98 (16)	C18—C19—H19A	109.4
C27—C22—N1	119.28 (16)	C20—C19—H19A	109.4
C23—C22—N1	120.73 (15)	C18—C19—H19B	109.4
O3—C21—N1	122.18 (16)	C20—C19—H19B	109.4
O3—C21—C14	121.21 (15)	H19A—C19—H19B	108.0
N1—C21—C14	116.56 (14)	C17—C18—C19	111.0 (2)
C25—C24—C23	120.89 (18)	C17—C18—H18A	109.4
C25—C24—H24	119.6	C19—C18—H18A	109.4
C23—C24—H24	119.6	C17—C18—H18B	109.4
O2—C11—C10	110.77 (15)	C19—C18—H18B	109.4
O2—C11—C14	101.78 (15)	H18A—C18—H18B	108.0
C10—C11—C14	119.79 (16)	C3—C2—C1	121.5 (3)
O2—C11—H11	108.0	C3—C2—H2	119.2
C10—C11—H11	108.0	C1—C2—H2	119.2
C14—C11—H11	108.0	C16—C20—C19	109.81 (19)
C21—C14—C13	114.24 (14)	C16—C20—H20A	109.7
C21—C14—C11	111.47 (15)	C19—C20—H20A	109.7
C13—C14—C11	100.47 (14)	C16—C20—H20B	109.7
C21—C14—C15	110.41 (14)	C19—C20—H20B	109.7
C13—C14—C15	103.12 (14)	H20A—C20—H20B	108.2
C11—C14—C15	116.61 (15)	C9—C10—C1	120.2 (2)
C24—C23—C22	118.81 (16)	C9—C10—C11	120.6 (2)
C24—C23—C13	122.87 (16)	C1—C10—C11	119.1 (2)
C22—C23—C13	118.32 (14)	C7—C6—C5	122.1 (3)
N2—C16—C20	109.22 (17)	C7—C6—C1	119.1 (3)
N2—C16—C15	103.46 (14)	C5—C6—C1	118.8 (3)
C20—C16—C15	117.31 (17)	C4—C5—C6	121.4 (3)
N2—C16—H16	108.8	C4—C5—H5	119.3
C20—C16—H16	108.8	C6—C5—H5	119.3
C15—C16—H16	108.8	C2—C3—C4	120.3 (4)
C26—C25—C24	119.69 (17)	C2—C3—H3	119.9
C26—C25—H25	120.2	C4—C3—H3	119.9
C24—C25—H25	120.2	C10—C9—C8	120.5 (3)
C25—C26—C27	120.44 (18)	C10—C9—H9	119.8
C25—C26—H26	119.8	C8—C9—H9	119.8
C27—C26—H26	119.8	C8—C7—C6	122.0 (2)
C16—C15—C14	105.51 (14)	C8—C7—H7	119.0
C16—C15—H15A	110.6	C6—C7—H7	119.0
C14—C15—H15A	110.6	C7—C8—C9	119.9 (3)
C16—C15—H15B	110.6	C7—C8—H8	120.0
C14—C15—H15B	110.6	C9—C8—H8	120.0
H15A—C15—H15B	108.8	C5—C4—C3	120.4 (3)
C26—C27—C22	120.18 (19)	C5—C4—H4	119.8
C26—C27—H27	119.9	C3—C4—H4	119.8
C22—C27—H27	119.9		
			
C17—N2—C13—C23	−60.8 (2)	C24—C25—C26—C27	0.4 (3)
C16—N2—C13—C23	169.99 (14)	N2—C16—C15—C14	16.5 (2)
C17—N2—C13—C14	175.55 (16)	C20—C16—C15—C14	136.84 (18)
C16—N2—C13—C14	46.34 (16)	C21—C14—C15—C16	−112.10 (16)
C17—N2—C13—C12	68.7 (2)	C13—C14—C15—C16	10.35 (19)
C16—N2—C13—C12	−60.50 (17)	C11—C14—C15—C16	119.34 (17)
C21—N1—C22—C27	168.63 (18)	C25—C26—C27—C22	0.0 (3)
C21—N1—C22—C23	−11.5 (3)	C23—C22—C27—C26	0.1 (3)
C22—N1—C21—O3	178.26 (18)	N1—C22—C27—C26	179.97 (18)
C22—N1—C21—C14	−4.4 (3)	C13—N2—C17—C18	173.85 (18)
C12—O2—C11—C10	−160.48 (17)	C16—N2—C17—C18	−60.7 (2)
C12—O2—C11—C14	−32.03 (19)	C11—O2—C12—O1	−175.29 (19)
O3—C21—C14—C13	−153.33 (17)	C11—O2—C12—C13	7.8 (2)
N1—C21—C14—C13	29.3 (2)	N2—C13—C12—O1	−48.6 (3)
O3—C21—C14—C11	93.6 (2)	C23—C13—C12—O1	83.0 (2)
N1—C21—C14—C11	−83.75 (19)	C14—C13—C12—O1	−157.0 (2)
O3—C21—C14—C15	−37.7 (2)	N2—C13—C12—O2	128.05 (16)
N1—C21—C14—C15	144.95 (16)	C23—C13—C12—O2	−100.40 (17)
N2—C13—C14—C21	85.93 (17)	C14—C13—C12—O2	19.64 (19)
C23—C13—C14—C21	−38.5 (2)	N2—C17—C18—C19	55.8 (3)
C12—C13—C14—C21	−156.33 (15)	C20—C19—C18—C17	−54.5 (3)
N2—C13—C14—C11	−154.64 (13)	C6—C1—C2—C3	2.6 (4)
C23—C13—C14—C11	80.93 (17)	C10—C1—C2—C3	−177.1 (2)
C12—C13—C14—C11	−36.89 (16)	N2—C16—C20—C19	−56.7 (2)
N2—C13—C14—C15	−33.92 (17)	C15—C16—C20—C19	−173.89 (19)
C23—C13—C14—C15	−158.35 (15)	C18—C19—C20—C16	54.5 (3)
C12—C13—C14—C15	83.83 (17)	C2—C1—C10—C9	179.6 (2)
O2—C11—C14—C21	164.11 (14)	C6—C1—C10—C9	−0.1 (3)
C10—C11—C14—C21	−73.4 (2)	C2—C1—C10—C11	−1.6 (3)
O2—C11—C14—C13	42.69 (16)	C6—C1—C10—C11	178.66 (19)
C10—C11—C14—C13	165.16 (18)	O2—C11—C10—C9	26.4 (3)
O2—C11—C14—C15	−67.84 (17)	C14—C11—C10—C9	−91.6 (3)
C10—C11—C14—C15	54.6 (2)	O2—C11—C10—C1	−152.35 (19)
C25—C24—C23—C22	0.9 (3)	C14—C11—C10—C1	89.7 (3)
C25—C24—C23—C13	−179.63 (18)	C2—C1—C6—C7	179.0 (2)
C27—C22—C23—C24	−0.5 (3)	C10—C1—C6—C7	−1.3 (3)
N1—C22—C23—C24	179.61 (17)	C2—C1—C6—C5	−1.9 (3)
C27—C22—C23—C13	179.96 (17)	C10—C1—C6—C5	177.8 (2)
N1—C22—C23—C13	0.1 (3)	C7—C6—C5—C4	179.1 (3)
N2—C13—C23—C24	87.6 (2)	C1—C6—C5—C4	0.0 (4)
C14—C13—C23—C24	−155.05 (17)	C1—C2—C3—C4	−1.5 (4)
C12—C13—C23—C24	−43.7 (2)	C1—C10—C9—C8	1.3 (4)
N2—C13—C23—C22	−92.97 (19)	C11—C10—C9—C8	−177.5 (3)
C14—C13—C23—C22	24.4 (2)	C5—C6—C7—C8	−177.6 (3)
C12—C13—C23—C22	135.75 (17)	C1—C6—C7—C8	1.4 (4)
C13—N2—C16—C20	−165.09 (15)	C6—C7—C8—C9	−0.3 (5)
C17—N2—C16—C20	61.9 (2)	C10—C9—C8—C7	−1.1 (5)
C13—N2—C16—C15	−39.42 (18)	C6—C5—C4—C3	1.3 (5)
C17—N2—C16—C15	−172.47 (17)	C2—C3—C4—C5	−0.6 (5)
C23—C24—C25—C26	−0.8 (3)		

### Hydrogen-bond geometry (Å, º)

**Table:**

*D*—H···*A*	*D*—H	H···*A*	*D*···*A*	*D*—H···*A*
N1—H1*A*···O3^i^	0.94 (3)	1.92 (3)	2.8413 (19)	167 (2)
C24—H24···O1^ii^	0.93	2.59	3.268 (2)	131

Symmetry codes: (i) −*x*, −*y*+1, −*z*+1; (ii) −*x*, −*y*, −*z*.

## References

[bb1] Bruker (2004). *APEX2*, *SAINT*, *XPREP* and *SADABS*. Bruker AXS Inc., Madison, Wisconsin, USA.

[bb2] Farrugia, L. J. (2012). *J. Appl. Cryst.* **45**, 849–854.

[bb3] Govindan, E., Yuvaraj, P. S., Reddy, B. S. R., Bangaru Sudarsan Alwar, S. & SubbiahPandi, A. (2014). *Acta Cryst.* E**70**, o168. 10.1107/S1600536814000130 PMC3998324 24764885

[bb4] Padwa, A., Brodney, M. A., Liu, B., Satake, K. & Wu, T. (1999). *J. Org. Chem.* **64**, 3595–3607. 10.1021/jo982453g 11674487

[bb5] Sheldrick, G. M. (2008). *Acta Cryst.* A**64**, 112–122. 10.1107/S0108767307043930 18156677

[bb6] Spek, A. L. (2009). *Acta Cryst.* D**65**, 148–155. 10.1107/S090744490804362X PMC2631630 19171970

