# Supplementary figures and images for: Crystal structure of 15-(naphthalen-1-yl)-7,7a,8,9,10,11-hexa­hydro-6a,12a-(methano­epoxy­methano)­indolizino[2,3-c]quinoline-6,13(5H)-dione
Source: Acta Crystallogr E Crystallogr Commun. 2015 Feb 7;71(Pt 3):o150–1. doi: 10.1107/S2056989015002017 (PMC4350735; doi:10.1107/S2056989015002017)

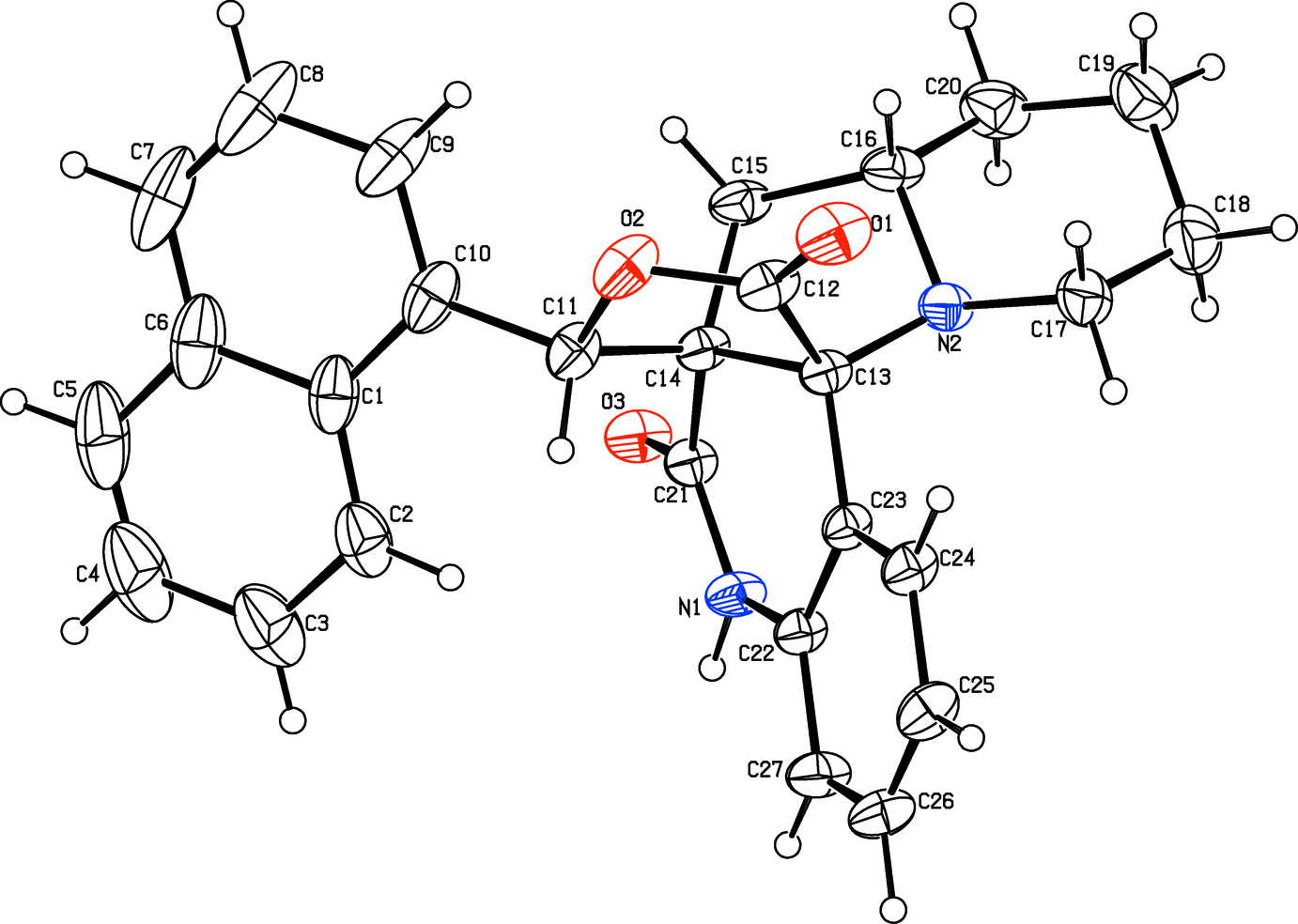

Supplement: Supplementary file 4 [file e-71-0o150-fig1.tif]

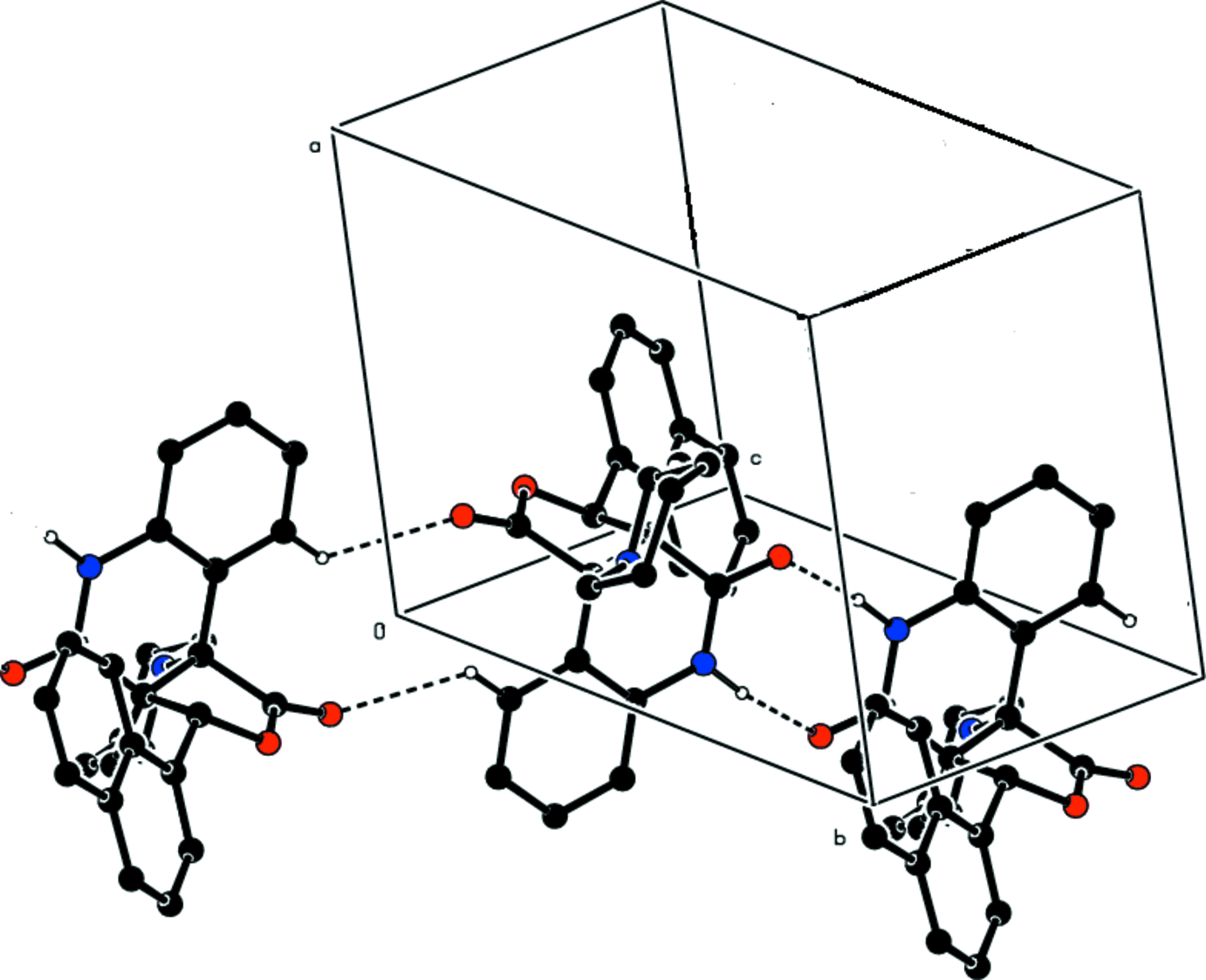

Supplement: Supplementary file 5 [file e-71-0o150-fig2.tif]

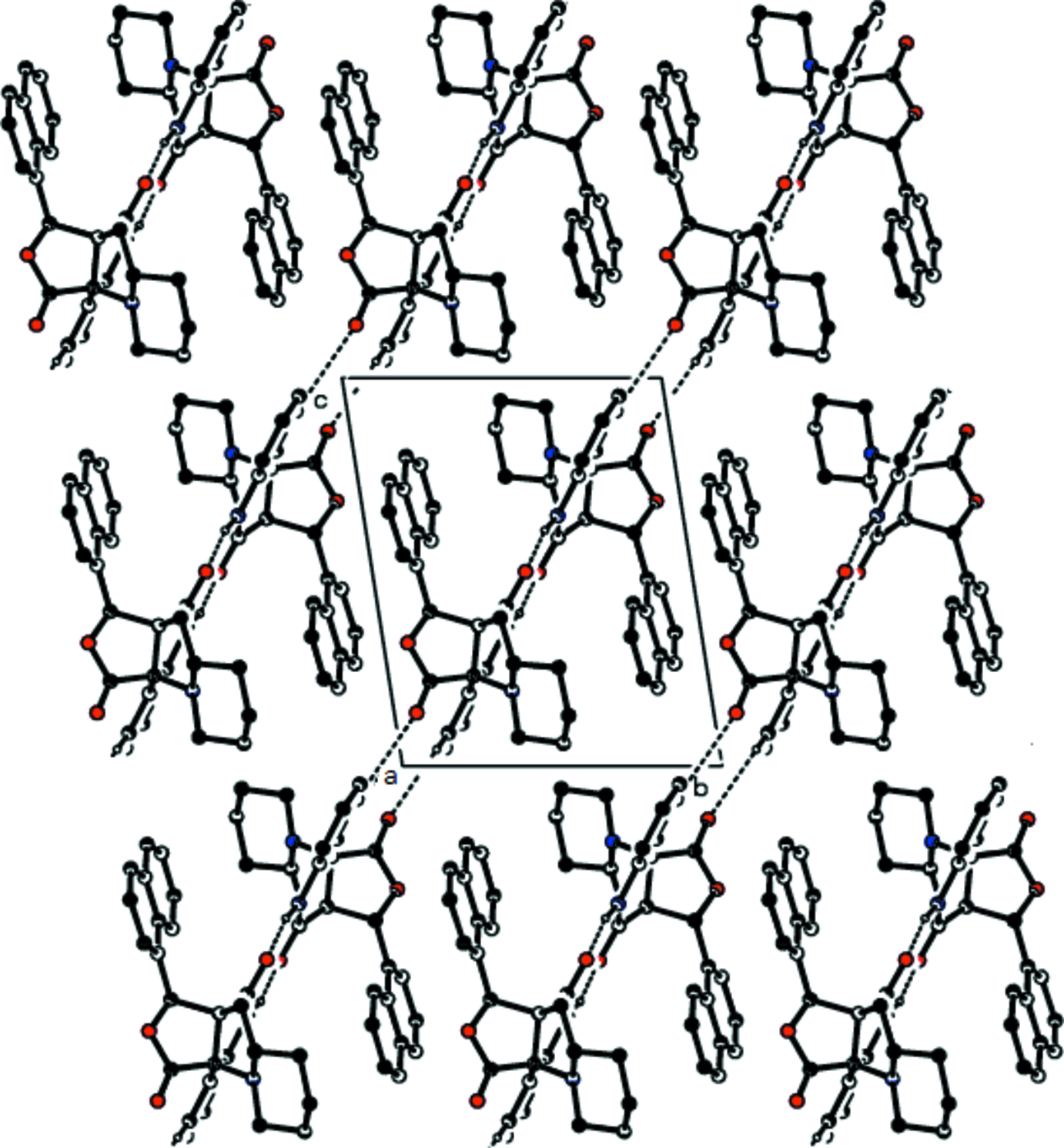

Supplement: Supplementary file 6 [file e-71-0o150-fig3.tif]
